# Double‐loop suture repair of radial meniscal tears provides favourable biomechanical performance compared to conventional repair techniques: A biomechanical study

**DOI:** 10.1002/jeo2.70366

**Published:** 2025-07-18

**Authors:** Antonio Petillo, Alessandro Di Rosa, Carmelo Burgio, Sofia Di Leonardo, Gaetano Burriesci, Francesco Bosco, Ludovico Lucenti, Lawrence Camarda

**Affiliations:** ^1^ Department of Precision Medicine in Medical, Surgical and Critical Care (Me.Pre.C.C.) University of Palermo Palermo Italy; ^2^ Adult Reconstruction and Joint Replacement Service Hospital for Special Surgery New York New York USA; ^3^ Department of Engineering University of Palermo Palermo Italy; ^4^ Department of Orthopaedics and Traumatology G.F. Ingrassia Hospital Unit Palermo Italy

**Keywords:** displacement, knee, load, meniscus, radial tear

## Abstract

**Purpose:**

Radial meniscal tears significantly impact knee biomechanics and can lead to joint degeneration if untreated. While various suture techniques exist, no consensus has been reached on the optimal method. The hypothesis was that the double‐loop suture can significantly reduce displacement between tear segments and achieve a higher ultimate failure load than conventional techniques. This study aims to biomechanically compare conventional repair techniques to a novel double‐loop suture to enhance tensile strength, reduce displacement and improve meniscal repair outcomes.

**Methods:**

Forty fresh‐frozen porcine menisci with full‐thickness radial tears were repaired using four techniques: double vertical, double horizontal, cross‐tie and double‐loop sutures. Biomechanical testing included cyclic loading (200 cycles, 5–19 N) and ultimate failure load evaluation. Key outcomes measured were displacement after cyclic loading, failure load, and failure modes. A one‐way analysis of variance (ANOVA) was conducted to identify significant differences among groups.

**Results:**

Among the evaluated techniques, the double‐loop suture demonstrated the highest ultimate failure load (111.1 ± 17.3 N, *p* < 0.01). Displacement after cyclic loading was minimal across techniques, except for the double vertical suture, which showed significantly higher displacement. Knot breakage was the primary failure mode in conventional sutures, whereas the double‐loop suture also exhibited tissue cutting.

**Conclusions:**

The double‐loop suture provides superior biomechanical performance, offering greater tensile strength and stability than conventional methods. Its simplicity and arthroscopic applicability highlight its potential for advanced meniscal repair. The double‐loop suture could be applied in the arthroscopic setting, improving the outcomes for treating radial tears. Further clinical studies are needed to confirm long‐term efficacy.

**Level of Evidence:**

Level IV, cadaveric study.

**Abbreviations**
FL: Florida
kN: kilonewton
MA: Massachusetts
mm: millimetre
N: Newton
NY: New York
SD: standard deviation
SPSS: Statistical Package for the Social Sciences
USA: United States of America

## INTRODUCTION

Radial tears of the meniscus, in particular, present unique challenges for orthopaedic surgeons due to the substantial tensile forces exerted on sutures during dynamic knee movements [1, 2, 6, 7, 13, 16, 24]. These injuries compromise the load transmission capacity of the meniscus, potentially leading to progressive joint degeneration if left inadequately treated. Clinical outcomes remain limited while various suture techniques have been proposed—such as double horizontal, double vertical, and cross‐tie patterns—and no consensus has been reached regarding the optimal approach. Among these techniques, the cross‐tie suture has been reported to demonstrate superior biomechanical properties; [10, 11, 13, 15] however, the quest for a method that consistently achieves long‐term success continues to drive research and innovation.

In response to these challenges, we have developed the double‐loop suture, a novel technique specifically designed to improve the biomechanical performance of meniscal repairs under weight‐bearing conditions. This approach incorporates strategic modifications to existing suture patterns, aiming to enhance tensile strength, minimise the risk of suture failure during knee movement, and create a stable environment that fosters optimal meniscal healing. By providing a more robust fixation of the tear, the double‐loop suture seeks to address the limitations of current techniques and improve patient outcomes.

A biomechanical evaluation is necessary to understand further how the different types of sutures can resist mechanical solicitation and, eventually, their role in reaching optimal clinical outcomes [3, 6, 17].

This experimental biomechanical study evaluates conventional meniscal suture techniques—specifically, the double vertical, double horizontal, and cross‐tie sutures—and compares their performance with the newly developed double‐loop sutures. This investigation assessed key mechanical properties under controlled laboratory conditions, such as reduced displacement at the tear site and increased ultimate failure load [3, 6, 17]. By addressing the limitations of existing techniques, the study aimed to provide a more effective surgical approach for repairing radial meniscal tears, ultimately contributing to improved long‐term patient outcomes.

We hypothesise that the double‐loop suture will significantly reduce displacement between tear segments and achieve a higher ultimate failure load than conventional techniques, offering an enhanced solution for repairing radial meniscal tears.

## METHODS

### Study design

This study was conducted as an experimental biomechanical evaluation to compare the mechanical performance of four meniscal suture techniques. A total of 40 fresh‐frozen porcine menisci were utilised, sourced from a local butcher. Initially processed for human consumption, these specimens did not require special ethical permissions for use in this investigation. The menisci were meticulously harvested from porcine tibiae, preserved at −20°C to maintain tissue integrity, and thawed at room temperature approximately 12 h before testing. All specimens were immersed in a saline solution and periodically misted with the same solution to prevent dehydration during the experimental procedures. Both medial and lateral menisci were included in the study and were randomly assigned to one of the four suture techniques under investigation. Randomisation was achieved using an online computer‐generated sequence to ensure an unbiased allocation of the samples (https://www.random.org/sequences/).

### Inclusion criteria

Stringent inclusion criteria were established to ensure the reliability of the biomechanical testing. Only menisci free from visible structural damage, deformation, or abnormalities during harvesting and preparation were included. Each specimen was inspected thoroughly to exclude those with tears, discoloration, or other irregularities that could compromise the biomechanical evaluation. This process was critical to standardise the testing conditions and reduce variability in the experimental outcomes.

### Surgical technique

Full‐thickness tears were created at the central portion of each meniscus using a No. 10 scalpel to simulate standardised radial meniscal injuries. The incisions were carefully positioned equidistant from the anterior and posterior horns to ensure uniform tear morphology across all specimens. Repairs were performed using non‐absorbable sutures (2‐0 FiberWire, Arthrex, North Naples, FL, USA) and surgical needles (B 204/13 Acufirm Wundnadeln, Germany). A single experienced orthopaedic surgeon (L.C.) performed all procedures to ensure consistency and reduce variability in applying the repair techniques. Four distinct suture methods were evaluated in this study, each selected for its clinical relevance and biomechanical potential: the double horizontal suture, cross‐tie suture, double vertical suture and the novel double‐loop suture.

**Figure 1:**
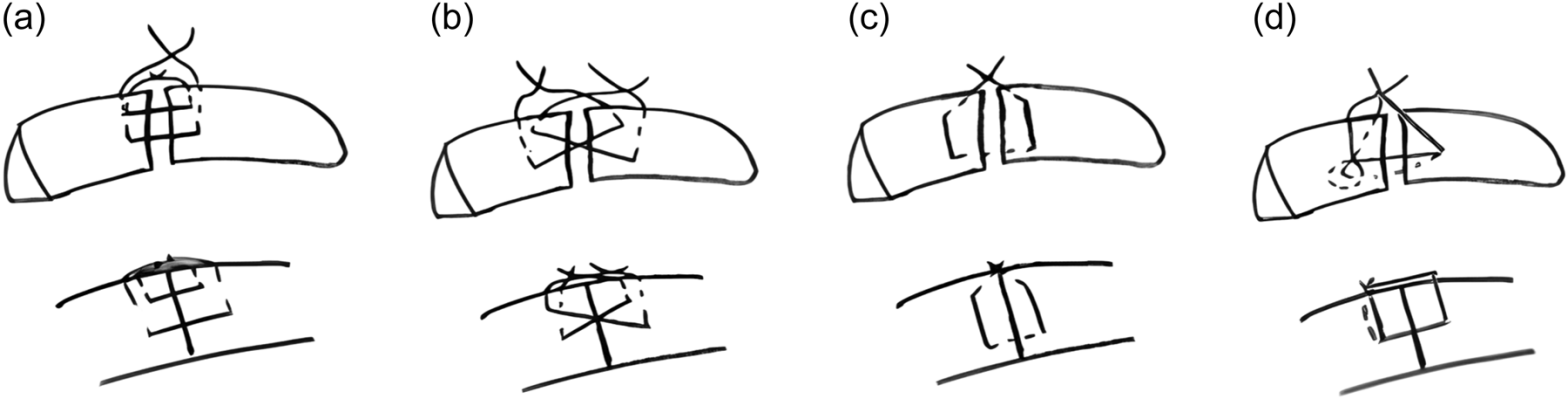
Schematic representation of the four suture techniques evaluated: (a) Double horizontal suture, (b) cross‐tie suture, (c) double vertical suture and (d) double‐loop suture.

The double horizontal suture employed two suture threads positioned parallel to the tear, with each thread passed through the meniscus 4 mm from the tear edges. These threads were tied externally in pairs, forming two parallel suture lines that enhanced contact between the tear margins. This technique offers increased stability and promotes healing by providing a broad compression area, which is especially beneficial in managing tears subjected to repetitive cyclic loading (Figure 1a).

The cross‐tie suture, described by Matsubara et al. [5], employed a crisscross pattern to stabilise the tear. The first suture thread was passed diagonally from the outer surface of the meniscus, approximately 5 mm from one edge of the tear to the inner surface on the opposite side. A second suture mirrored this pattern in the opposite direction, forming an 'X' configuration over the tear. Knots were tied on the external surface to secure the repair. This technique was chosen for its ability to distribute tensile forces evenly across the tear, and it reported superior biomechanical performance in minimising displacement during repetitive loading (Figure 1b).

The double vertical suture technique involved vertically passing a single suture thread from the exterior surface of the meniscus, approximately 4 mm from the tear, into the joint space, then through the meniscal tissue to the interior surface, and back out to the exterior surface on the opposite side of the tear. The knot was securely tied on the external surface of the meniscus, creating compression across the tear site. This technique is commonly utilised in clinical practice due to its simplicity and ability to provide stable fixation, particularly for vertical tear configurations (Figure 1c).

**Figure 2:**
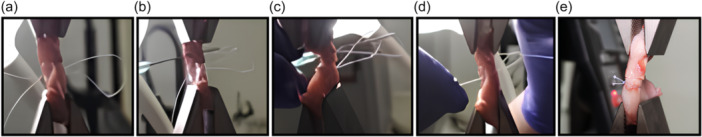
Stepwise execution of the double‐loop suture technique on fresh‐frozen porcine menisci. (a) Passage of the first needle through the meniscus, creating the initial loop on the inner surface of the tear. (b) Passage of the second needle, forming a second loop on the opposite side of the tear. (c) The second loop is threaded through the first loop, securing the construct in place. (d) Tension is applied to the suture by pulling the thread of the second loop, ensuring tight approximation of the tear edges. (e) Finalisation of the repair with the external knot tying after the free suture end is pulled through, securing the meniscal tear.

**Figure 3:**
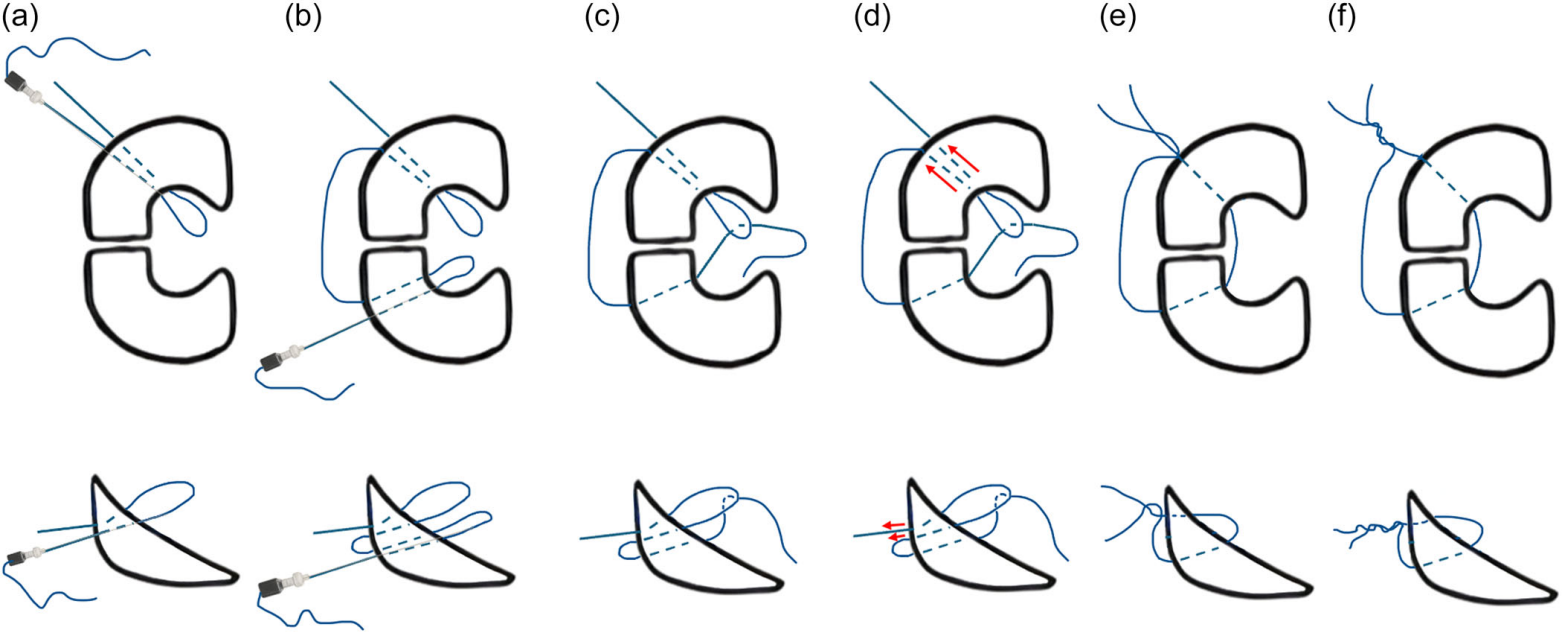
Schematic representation of the double‐loop suture technique for radial meniscal tear repair. (a) A first suture limb is passed through the inner edge of the radial tear, creating the initial loop on the inner meniscal surface. (b) A second pass is made on the opposite side of the tear, forming a second loop on the outer side of the radial defect. (c) The second loop is threaded through the first loop, interlocking the two suture limbs. (d) The first suture limb is pulled to draw the second loop through the meniscal tissue, creating circumferential compression (red arrows indicate direction of traction). (e) Both suture limbs are now tensioned externally to approximate the tear edges and ensure stability of the repair. (f) Final knot tying is performed on the capsular side, securing the repair construct.

The double‐loop suture technique was performed using a single suture thread passed through the meniscus with a syringe needle. Initially, a loop was created on one side of the radial tear by passing the suture from the outer capsular surface to the inner meniscal aspect. The same suture was then passed on the opposite side of the tear to create a second loop. This second loop was threaded through the first, ensuring interlocking of the construct. Subsequently, the free ends of the suture were pulled externally to approximate the tear edges, and final fixation was achieved by tying three secure knots on the outer surface. This configuration enhanced tensile strength and reduced displacement by improving contact stability across the tear site (Figures 1d, 2, and 3).

All four suture techniques were performed using an inside‐out approach, with the final knots consistently tied on the external surface of the meniscus to standardise the repair. These techniques were selected based on their clinical relevance and potential biomechanical benefits, and their execution was carefully controlled to ensure uniformity across all specimens.

### Definition of study outcomes

Data collection focused on two primary parameters: displacement and ultimate failure load. Displacement was defined as the difference between the maximum elongation recorded during the first loading cycle and the maximum elongation recorded during the 200th loading cycle under cyclic loading conditions. Ultimate failure load was defined as the peak force applied to the meniscal repair before a catastrophic failure occurred, as indicated by a sudden drop in force during testing. Additionally, the failure mode of each specimen, such as suture breakage, meniscal tear extension, or suture pullout, was carefully documented to provide a comprehensive understanding of the mechanical performance of each repair technique.

### Testing protocol

Biomechanical testing was conducted to evaluate the mechanical performance of the four suture techniques under standardised conditions that simulated the physiological forces experienced by menisci during normal knee joint function. All tests were performed using a high‐precision electromechanical testing machine (Model 5943, Instron, Canton, MA, USA) equipped with a 1‐kN load cell to measure both force and displacement accurately. The menisci were securely mounted between two universal grips with roughened interior surfaces designed to maximise grip strength without damaging the tissue to prevent slippage and ensure consistent positioning of the specimens.

The testing protocol was divided into three phases: preloading, cyclic loading, and failure testing. The preloading phase was designed to eliminate slack in the sutures and to precondition the meniscal tissue, mimicking the initial stabilisation that occurs in vivo after surgical repair. The choice of N applied was based on previous studies. In particular, the selection of 5 N as the preloading force was related to the absolute maximum probing force used in the most accurate research conducted in the literature for the push task of 2.8 N (±0.8) N, for the continuous run task of 2.5 N (±0.9) N, and for the pull task of 3.9 (±2.0) N [8, 23, 24]. During this phase, a controlled force of up to 5 N was applied at a rate of 6 N/min. The specimens were then stabilised under a constant load of 5 N for 60 s to ensure uniform starting conditions for all subsequent tests.

The second phase, cyclic loading, aimed to simulate repetitive stress experienced by the meniscus under physiological conditions, such as walking or low‐impact activity. Each specimen was subjected to 200 loading cycles, with forces oscillating between 5 N and 19 N, under displacement control at a 100 mm/min speed. This phase assessed the durability of the suture techniques and the ability of the repairs to withstand repeated stress without significant displacement or structural failure. The displacement was recorded as the difference between the maximum elongation measured during the first cycle and the maximum elongation recorded during the 200th cycle.

The final phase evaluated the ultimate failure load, representing the maximum force the meniscal repair could sustain before catastrophic failure. Each specimen was subjected to continuous loading at a displacement‐controlled speed of 12.5 mm/min until failure occurred. Failure was defined as a sudden drop in force on the load‐displacement curve, accompanied by visible disruption of the meniscal repair. The ultimate failure load was recorded as the peak force applied before this event.

**Figure 4:**
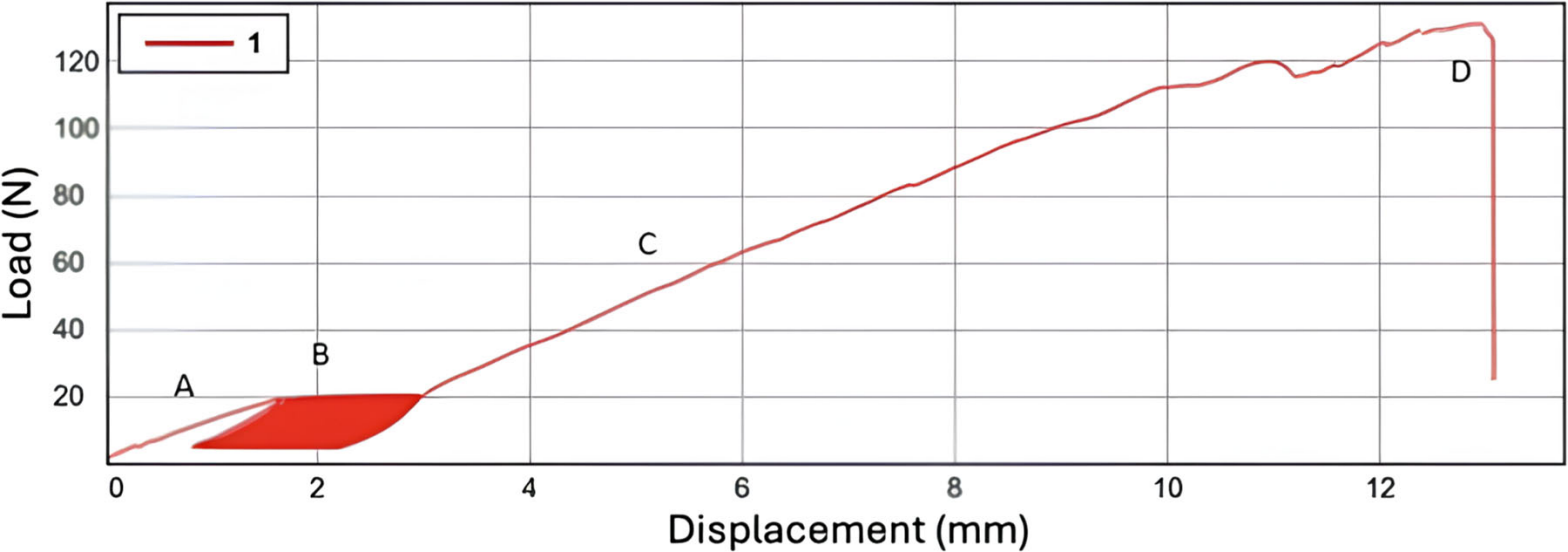
Representative load‐displacement curve from biomechanical testing of meniscal suture repairs. A. Preloading phase: A controlled force of up to 5 N is applied to eliminate slack and precondition the meniscal tissue. B. Cyclic loading phase: The specimen undergoes 200 cycles of repetitive loading between 5 N and 19 N, simulating physiological stresses encountered in vivo. The shaded red area represents the accumulated energy dissipation during cyclic loading. C. Progressive loading phase: The load increases steadily under displacement control, evaluating the mechanical stability and resistance of the repair technique. D. Failure point: The ultimate failure load is reached, characterised by a sudden drop in force, indicating structural failure of the repair (e.g., suture breakage, tear extension, or suture pullout). mm, millimetre; N, Newton.

Throughout the testing protocol, load‐displacement data were continuously recorded to provide detailed mechanical profiles for each suture technique. Representative load‐displacement curves, highlighting the performance of the four techniques during both cyclic loading and failure testing, are shown in Figure 4. In addition, the failure mode of each specimen was documented, noting whether failure occurred due to suture breakage, tear extension, or suture pullout.

This comprehensive protocol ensured that the mechanical properties of each suture technique, including displacement under cyclic loading and ultimate failure load, were rigorously assessed and directly comparable under standardised conditions.

### Statistical analysis

Statistical analyses were performed using SPSS software (version 27.0, IBM, Armonk, NY, USA) to compare the biomechanical performance of the four suture techniques regarding displacement and ultimate failure load. The normality of the data was assessed with the Shapiro–Wilk test, and the homogeneity of variances was verified using Levene's test. A one‐way analysis of variance (ANOVA) was conducted to identify significant differences among groups. When significant, Tukey's Honestly Significant Difference (HSD) test was used for post‐hoc comparisons, maintaining a significance threshold of *p* < 0.05. In doing multiple comparisons in small sample sizes, Tukey's test is robust while still controlling for false positives.

Effect sizes were calculated using partial eta‐squared (*η*²) to quantify the magnitude of group differences, with thresholds of 0.01, 0.06 and 0.14 interpreted as small, moderate, and large, respectively. Descriptive statistics were employed to analyse failure modes (e.g., suture breakage, tear propagation or pullout) as percentages within each group. Sample size adequacy was determined a priori using GPower software (version 3.1). Based on an alpha level of 0.05, a power of 80%, and an anticipated effect size of 0.40, a minimum of eight specimens per group was required. Results are expressed as mean ± standard deviation (SD). The anticipated effect size of 0.40 refers to a moderate effect based on Cohen's guidelines for interpreting effect sizes. This value suggests a moderate difference between groups.

## RESULTS

**Figure 5:**
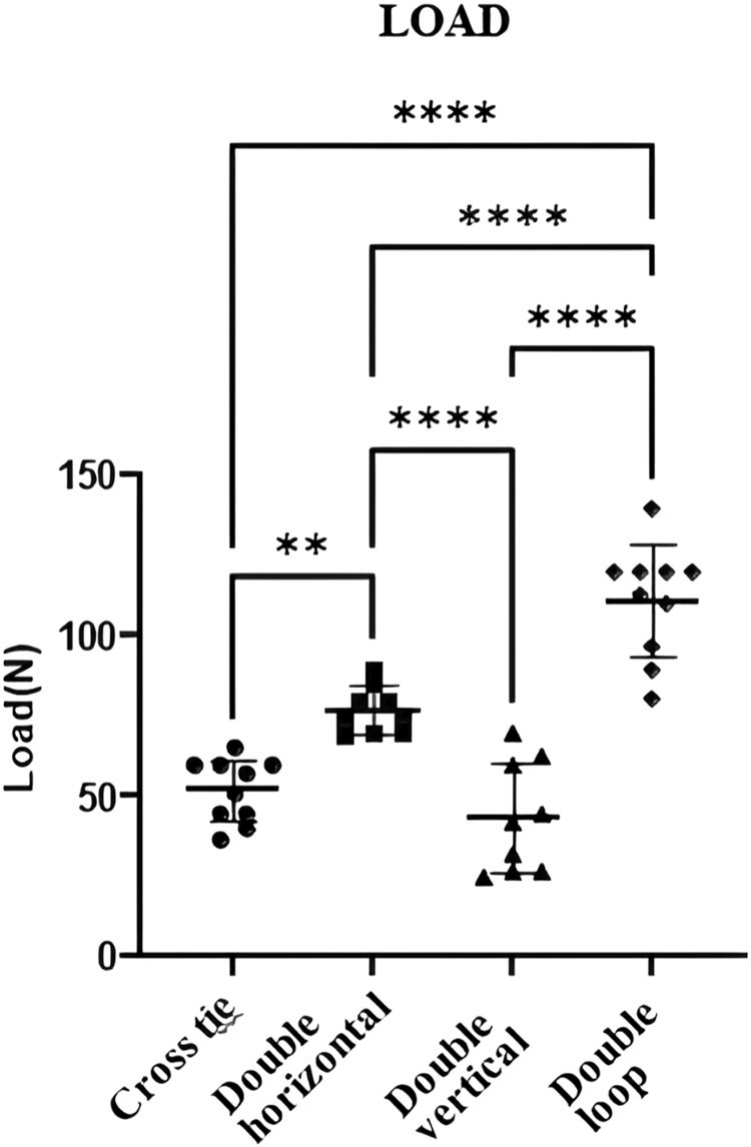
Comparison of ultimate failure load across the four suture techniques. N, Newton.

All 40 specimens were successfully tested without exclusions or dropouts. The double‐loop suture demonstrated superior ultimate failure load than the other techniques, with a mean value of 111.1 ( ± 17.3) N. This was significantly higher than the ultimate failure load observed in the double horizontal suture: 77 ( ± 7.3 N), *p* < 0.01. No significant difference was detected between the cross‐tie and double vertical sutures (*p* = 0.5). Similarly, the ultimate failure load of the double horizontal suture was significantly lower than that of the double‐loop suture (*p* < 0.01), confirming the enhanced tensile strength of the latter technique (Table 1 and Figure 5).

**Table 1:** Mean ultimate load to failure and displacement values for each suture technique.

Suture techniques	Load, *n* (mean ± SD)	Displacement, mm (mean ± SD)
Cross‐tie	52 ± 9.7	0.95 ± 0.4
Double horizontal	77 ± 7.3	0.8 ± 0.2
Double vertical	43.4 ± 17.2	1.9 ± 0.1
Double‐loop*	111.1 ± 17.3	1.2 ± 0.4

Abbreviations: mm, millimetre; N, Newton; SD, standard deviation.

* In the double‐loop group, 3/10 failures occurred via suture breakage and 7/10 via tissue cutting (*p* = 0.206).

**Figure 6:**
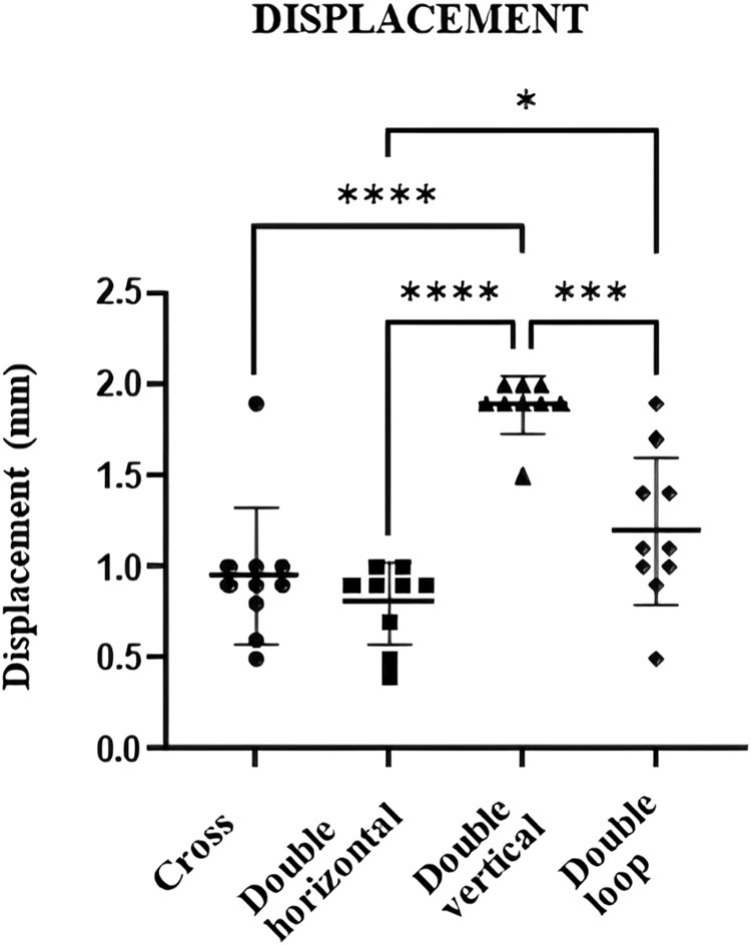
Comparison of displacement after 200 loading cycles for the four suture techniques. mm, millimetre.

Displacement after 200 cycles also varied significantly across the techniques. The mean value of the cross‐tie suture of 0.95 ( ± 0.4) mm and the double horizontal suture of 0.8 ± (0.2) mm demonstrated smaller displacement values compared to the double vertical and double‐loop sutures, indicating superior stability under repetitive loading (*p* < 0.01). While no significant difference was found between the cross‐tie and double horizontal sutures, the displacement recorded for the double‐loop suture was minimal but significantly lower than that of the double vertical suture (*p* < 0.01). These results highlight the mechanical robustness of the cross‐tie and double horizontal techniques in maintaining tear stability under cyclic loading conditions (Table 1 and Figure 6).

Failure mode analysis provided additional insights into the mechanical performance of the suture techniques. The predominant failure mode for the cross‐tie, double horizontal, and double vertical suture groups was thread breakage near the knot, accounting for most instances of failure. Conversely, two distinct failure modes were identified in the double‐loop suture group. In three specimens, failure occurred due to thread breakage near the knot. In seven specimens, failure occurred due to suture‐induced tissue cutting, suggesting a trade‐off between tensile strength and tissue integrity. These findings underscore the unique biomechanical behaviour of the double‐loop suture compared to conventional techniques.

In the double‐loop suture group, a suture breakage was noticed in 3 out of 10 specimens (30%), while a tissue cutting was verified in 7 out of 10 specimens (70%), *p* 0.206. This proportion of tissue cutting suggests a trend of failure mode that is more common in the double‐loop suture technique (Table 1 and Figures 5 and 6).

## DISCUSSION

The main finding of this study is that the novel double‐loop suture technique demonstrated superior biomechanical performance compared to the established double horizontal, cross‐tie, and double vertical sutures for repairing radial meniscal tears. The study supported the hypothesis that the double‐loop suture could achieve a higher ultimate failure load than conventional techniques. The double‐loop suture also maintained minimal displacement after 200 loading cycles, comparable to other stable configurations. These findings establish the double‐loop technique as a potentially superior solution for addressing the biomechanical challenges of radial meniscal tears.

Radial meniscal tears, historically considered irreparable, were often managed with meniscectomy, which is associated with a higher risk of early osteoarthritis due to altered joint biomechanics [3, 13, 14, 20]. Over the past two decades, the emphasis has shifted toward preserving the meniscal structure to maintain its critical roles in load distribution, shock absorption, and joint stability. This shift has driven the development of advanced suture techniques to restore meniscal integrity while maintaining its functional properties. Despite these advancements, many current techniques exhibit tensile strength and stability limitations, as evidenced by suboptimal clinical and biomechanical outcomes [3, 4, 9, 10, 11, 12, 19, 20, 25].

The double‐loop suture introduced in this study demonstrated a unique design advantage that contributed to its superior performance. By forming two loops around the tear, this technique allows for a more even distribution of tensile forces, reducing the risk of knot‐related failures while enhancing the stabilisation of the tear. This configuration also minimises displacement under cyclic loading, a key factor in promoting effective healing by maintaining tear edge apposition [5]. Moreover, the double‐loop suture does not require specialised instruments, making it adaptable to diverse clinical settings, including arthroscopic applications in challenging zones such as the posterior middle region of the meniscus [4, 22].

Despite its widespread use, the double horizontal suture exhibited lower tensile strength (77 ± 7.3 N) and a limited ultimate failure load. While the design offers moderate stability through parallel suture lines, it falls short in effectively managing high‐tensile forces compared to the double‐loop suture. This limitation underscores the need for improved techniques to repair radial meniscal tears to enhance overall knee stability and functionality. Similarly, the cross‐tie suture, known for its enhanced stability due to its crisscross pattern, performed better in displacement resistance but exhibited lower ultimate failure load (52 ± 9.7 N), highlighting a trade‐off between stability and tensile strength. The double vertical suture, with the lowest ultimate failure load (43.4 ± 17.2 N), appears less effective in redistributing forces across the tear, limiting its utility in radial meniscal repairs.

Failure mode analysis revealed critical insights into the mechanical behaviour of these techniques. In the cross‐tie, double horizontal, and double vertical sutures, thread breakage near the knot was the predominant failure mechanism, reflecting the mechanical limitations of these configurations under high tensile stress. In contrast, the double‐loop suture exhibited two distinct failure patterns: thread breakage near the knot in three specimens and suture cutting through the meniscal tissue in seven specimens. The latter failure mode suggests that while the double‐loop suture offers superior tensile strength, the concentrated forces on the meniscal tissue may increase the risk of tissue damage. However, this trade‐off may be clinically acceptable, particularly in cases where enhanced tear stabilisation is critical for preventing progressive degeneration [5, 8].

The entity and severity of cutting, resulting from high localised stress at the suture‐tissue interface, was not quantified in detail, making it unclear whether this damage is superficial or deep enough to compromise meniscal integrity. The risk of tissue cutting may alter the long‐term viability of meniscal repairs using the double‐loop technique.

The clinical significance of these findings lies in the potential for the double‐loop suture to address the limitations of current techniques, particularly in high‐demand patients or challenging tear locations. On the other hand, biomechanical properties do not necessarily result in better clinical outcomes. The risk of re‐tear, particularly under dynamic loading conditions (weight‐bearing and/or knee flexion‐extension cycles) should be considered.

Previous clinical studies, such as those by Tsujii et al., have reported high partial or total healing rates following suture repair of radial meniscal tears. However, these studies also highlighted the progression of chondral lesions, emphasising the need for techniques to provide more substantial and more stable repairs [22]. The double‐loop suture's enhanced tensile strength and minimal displacement suggest that it could mitigate these issues by providing better tear stabilisation during the critical healing phase.

Some strategies can be used to limit tissue cutting in clinical practice and to optimise the sutures. Using wider sutures with a protective coating or softer texture may help distribute force over the surface area and reduce stress concentration. Furthermore, over‐tightening may exacerbate tissue cutting by increasing localised compression. Avoiding excessive stress on the meniscus, placing sutures along the strongest collagen fibre orientation, and creating alternative configurations (reinforcing sutures or hybrid techniques) should be employed to ensure stability.

The double‐loop suture could be applied in the arthroscopic setting, improving the outcomes for treating radial tears. Rehabilitation may also play a role: controlled motion protocols and gradual loading progression may reduce excessive mechanical stress on the repair site and prevent premature mechanical failure.

This study offers several notable strengths. First, it provides a rigorous biomechanical comparison of a novel suture technique against established methods under standardised conditions, yielding robust and reproducible data. Second, the double‐loop suture was explicitly designed with practical applicability in mind, combining biomechanical efficiency with ease of execution and adaptability to arthroscopic procedures. Third, the comprehensive testing protocol, including cyclic loading and ultimate failure testing, provides valuable insights into the durability and performance of each technique under extreme conditions.

Despite its strengths, the study has limitations that should be acknowledged. The use of porcine menisci, while anatomically like human menisci, may not fully replicate the biomechanical properties of human tissue due to differences in cartilage density and thickness [3, 11, 13, 26]. Even if fresh‐frozen porcine menisci for biomechanical testing provides a reasonable model, some differences in biomechanical properties can be detected. Variations in cartilage density, thickness, and collagen fibre orientation among the different areas of the meniscus of different species may influence load distribution and deformation under mechanical stress.

Porcine menisci are structurally like human menisci but show differences in size, shape, and thickness other than in the collagen fibre arrangement, extracellular matrix composition, cell density, and phenotype.

Furthermore, variations in proteoglycan and collagen content, different tensile and compressive properties, and the relative proportions of the medial and lateral menisci differ between species, affecting biomechanical function. All these factors may impact healing potential and response to treatments and potentially affect the clinical relevance of the findings [17, 18, 21].

Additionally, the testing protocol applied tangential distraction forces, which, although severe, do not entirely reflect the complex multidirectional stresses experienced by the meniscus in vivo [8, 26]. Another limitation is the controlled environment in which sutures were performed. In contrast, sutures were executed under direct observation for accuracy, and clinical repairs are typically performed arthroscopically within an intact joint capsule, which introduces additional challenges not replicated in this study. Future investigations should aim to validate these findings in clinical settings to assess the real‐world applicability of the double‐loop suture.

## CONCLUSION

This study demonstrated that the novel double‐loop suture technique offers superior biomechanical performance compared to established methods for repairing radial meniscal tears. With the highest ultimate failure load and minimal displacement under cyclic loading, the double‐loop suture provides enhanced stability and tensile strength, addressing key limitations of conventional techniques. Its straightforward execution and adaptability to arthroscopic settings further enhance its clinical applicability, particularly in challenging zones of the meniscus. The double‐loop suture could be applied in the arthroscopic setting and potentially improve the outcomes for treating radial tears. While these findings underscore the potential of the double‐loop suture to improve repair outcomes and promote meniscal preservation, future clinical studies are needed to validate its efficacy in vivo and assess its long‐term impact on knee joint function and patient outcomes.

## AUTHOR CONTRIBUTIONS

Antonio Petillo, Carmelo Burgio and Alessandro Di Rosa have contributed substantially to conception and design, data acquisition, analysis and interpretation. They agree to be accountable for all aspects of the work in ensuring that questions related to the accuracy or integrity of any part of the work are appropriately investigated and resolved. Sofia Di Leonardo and Gaetano Burriesci have contributed substantially to the data analysis, data acquisition and analysis. Francesco Bosco, Ludovico Lucenti, and Lawrence Camarda have significantly contributed to revising the manuscript critically for important intellectual content, giving final approval of the version to be published.

## CONFLICT OF INTEREST STATEMENT

The authors declare no conflicts of interest.

## ETHICS STATEMENT

This study was conducted in accordance with the ethical standards of the institutional and/or national research committee and with the 1964 Helsinki Declaration and its later amendments or comparable ethical standards.

## Data Availability

The data set analysed in this study is available from the corresponding author on reasonable request.
